# Reference Dosimetry according to the New German Protocol DIN 6800-2 and Comparison with IAEA TRS 398 and AAPM TG 51^*^

**DOI:** 10.2349/biij.7.2.e15

**Published:** 2011-04-01

**Authors:** A Zakaria, W Schuette, C Younan

**Affiliations:** Department of Medical Radiation Physics, Kreiskrankenhaus Gummersbach, Academic Teaching Hospital of the University of Cologne, Germany

**Keywords:** Absorbed dose to water, DIN 6800-2 (2008 March), IAEA TRS 398 (2000), AAPM TG-51 (1999)

## Abstract

The preceding DIN 6800-2 (1997) protocol has been revised by a German task group and its latest version was published in March 2008 as the national standard dosimetry protocol DIN 6800-2 (2008 March). Since then, in Germany the determination of absorbed dose to water for high-energy photon and electron beams has to be performed according to this new German dosimetry protocol. The IAEA Code of Practice TRS 398 (2000) and the AAPM TG-51 are the two main protocols applied internationally. The new German version has widely adapted the methodology and dosimetric data of TRS-398. This paper investigates systematically the DIN 6800-2 protocol and compares it with the procedures and results obtained by using the international protocols. The investigation was performed with 6 MV and 18 MV photon beams as well as with electron beams from 5 MeV to 21 MeV. While only cylindrical chambers were used for photon beams, the measurements of electron beams were performed by using cylindrical and plane-parallel chambers. It was found that the discrepancies in the determination of absorbed dose to water among the three protocols were 0.23% for photon beams and 1.2% for electron beams. The determination of water absorbed dose was also checked by a national audit procedure using TLDs. The comparison between the measurements following the DIN 6800-2 protocol and the TLD audit-procedure confirmed a difference of less than 2%. The advantage of the new German protocol DIN 6800-2 lies in the renouncement on the cross calibration procedure as well as its clear presentation of formulas and parameters. In the past, the different protocols evoluted differently from time to time. Fortunately today, a good convergence has been obtained in concepts and methods.

## INTRODUCTION

In Germany, the dosimetry for linear accelerators is currently performed according to the new DIN 6800-2 (2008 March) [1]. In this protocol, the absorbed dose in water is used as a measuring quantity for high-energy photon and electron beams. In order to better meet international consistency, the preceding DIN 6800-2(1997) [2] was modified and adapted to the Technical Report Series No. 398 (TRS 398) of the IAEA [3]. The description of the measurements and methods, the correction parameters for various chambers and the evaluation for uncertainties are the main parts of DIN 6800-2 (2008 March). The physical basics are illustrated in its appendix. The new data of the correction factors and the interaction coefficients of the IAEA TRS 398 are taken for the calculation of the absorbed dose in the new DIN, so that the international consistence of the dose calculation is assured. Although not all parameters were taken directly from TRS 398, the effects of the deviations from the TRS 398 remain low [4]. The main difference between the old protocol and the new one lies in the electron dosimetry as well as in the evaluation of the uncertainties. In this work, the authors have applied the new DIN 6800-2 (2008 March) to check the clinical dosimetry in reference to the TRS 398 (2000) as the base for the future application of the DIN 6800-2. Furthermore, the authors also included the protocol AAPM TG-51 (1999) [5] in the comparison. Although TRS 398 is aiming at being a “Code of Practice” rather than a dosimetry protocol in its real meaning, it will also be called a dosimetry protocol along with the two other documents in this manuscript.

## MATERIALS AND METHODS

The measurements for the evaluation of the depth dose distributions were performed in a Wellhoefer water phantom (Blue Phantom) with the plane-parallel Roos chamber 34001 (PTW). The cylindrical chamber CC-13 (Wellhoefer) was used as a reference chamber. The relative measurements were done and evaluated with the Software Scanditronix-Wellhöfer OmniPro-Accept 6.3.

The determination of the absorbed dose in water was also done in the Blue Phantom with two cylindrical chambers (PTW-31013 type) as well as with two plane parallel chambers (PTW-34001 type, Roos-Chamber).

For the absolute dosimetry, the Scanditronix-Wellhoefer-Assembly system was used only for the positioning of the measuring chamber. The absolute charge was measured with the electrometer models PTW-UNIDOS and PTW-UNIDOS E. Positioning of the ionisation chamber was performed by adjusting the reference point of a specific chamber to the measuring depth as required in the respective protocol (Table 1). The reference point of the cylindrical chamber is located at the central axis and at the middle point of the chamber cavity (rcyl = internal radius of the chamber cavity). For the Roos-chamber, the reference point is located on the inner surface of the entrance window which is at the centre of the window (PMMA thickness of the window: 1 mm). Each chamber type is calibrated in a Co-60 radiation beam with its reference point at measuring depth.

**Table 1:** Positioning of ionization chamber type according to the protocols.

**Chamber Type**	**IAEA TRS 398 (2000)**		**DIN 6800-2 (2008 March)**		**AAPM TG-51 (1999)**	
	**Photon**	**Electron**	**Photon**	**Electron**	**Photon**	**Electron**
**cylindrical-chamber**	Reference point at the measuring depth	Reference point 0.5 *r_cyl_* below the measuring depth	Reference point 0.5 *r_cyl_* below the measuring depth	Reference point 0.5 *r_cyl_* below the measuring depth	Reference point at the measuring depth	Reference point at the measuring depth
**Plane- parallel chamber**	-	Reference point at the measuring depth	-	Reference point at the measuring depth	-	Reference point at the measuring depth

The operating voltage for the cylindrical chamber is 400 V, whereas the Roos-chamber is connected to a voltage of 200 V. Furthermore, additional measurements were performed in a special type of water phantom PTW 4322 with TLD-dosimeters according to an audit procedure called technical control measurement (MTK) of the German medical products law (LMKM). The results were taken to additionally check the accuracy of this work.

Two linear accelerators, a Siemens ONCOR Impression (with 6 MV-Photon and 5, 7, 8, 10, 12, 14 MeV-Electron beams) and a Siemens ONCOR Avantgarde (with 6 and 18 MV-Photon and 6, 9, 12, 15, 18, 21 MeV-Electron beams) were available as irradiation units in the department for this study.

### Principle of the determination of the absorbed dose in water

All ionisation chambers were calibrated with a Co-60 source (and thus provided with calibration factor *N*). For the determination of the absorbed dose in water *D_w_* for photon and electron beams, having the dosimeter reading *M* at the depth of measurement *z_ref_*, the following equation is generally applied to all protocols as:

(1) $$D=M\cdot Ν\cdot \overset{n}{\underset{i=\text{1}}{\Pi }}k_{i}$$

where *N* – as mentioned – is the calibration factor for absorbed dose to water for Co-60 beam.

The product of the correction factors Π *k_i_* contains the required corrections to take into account all the deviations between the calibration and the measuring conditions for the determination of the absorbed dose in water. In all protocols, the most important corrections are those for the polarity of the chamber voltage (*k*_P_), for the ion recombination correction (*k*_S_), for the air density effect (*k*_ρ_) and for the radiation quality *k*_Q_ for photons and *k*_E_ for electrons, respectively. In the DIN-Protocol, an additional correction factor *k*_r_ is included for the cylindrical chamber, which takes explicitly into account the different position of cylindrical chambers during the calibration (reference point at measuring depth) and the user’s measurement (reference point 0.5 *r_cyl_* lower than measuring depth). In the other protocols, this correction factor is embedded in the quality correction factor *k*_Q_ as one of the constituent perturbation factors.

The reference conditions for the determination of the absorbed dose to water are listed in Table 2.

**Table 2:** Reference conditions for the determination of depth dose curve and the absorbed dose to water for all 3 protocols (IAEA suggests a field size FS = 10 × 10 cm^2^ for Electron beams, we have chosen the values for IAEA FS = 20 × 20 cm^2^ for all protocols for comparison).

	**Dose depth distribution**		**Absorbed dose to water**	
	**Photons**	**Electrons**	**Photons**	**Electrons**
**Reference-Field size**	10 × 10 cm^2^	20 × 20 cm^2^	10 × 10 cm^2^	20 × 20 cm^2^
**SSD**	100 cm	100 cm	100 cm	100 cm
**Reference depth**	-	-	*z_ref_* = 10 cm	*z_ref_* = 0.6*R_50_ − 0.1 [cm]

### Determination of correction factors

#### Determination of the correction factor *k*_ρ_ for the air density

*k*_ρ_ is calculated from the temperature in the phantom and the absolute pressure at the measuring place as follows:

(2) $$k_{\rho }=\frac{p_{0}T}{pT_{0}}$$

With *T* = temperature in the phantom, *p* = air pressure in place as well as the corresponding reference values of temperature (*T*_0_ = 293.2 K) and pressure (*p*_0_ = 1013.25 hPa).

All chambers are calibrated at 20°C in Germany. Therefore, the authors have also used 20°C for the calculation of absorbed dose for the AAPM- protocol instead of the temperature 22°C given there.

#### Determination of the correction factor *k*_S_ for the ion recombination

*k*_S_ can be determined either experimentally by the “two-voltage-procedure” or theoretically by a formula. The different methods are presented in Table 3. The authors have applied the experimental method. The difference between the experimental method and the theoretical calculation is smaller than 0.2% for all chambers and energies.

**Table 3:** Determination of the correction factor *k_s_* (where D_i_ = Dose per radiation pulse in mGy, U_1_ = normal chamber operating voltage, U_2_ = lower chamber voltage, d = Electrode distance, M_1_ = measured value at U_1_, M_2_ = measured value at U_2_, the constants γ and δ are to be taken form the DIN 6800-2 [Edition version: 2007], Table 4. The constants a_0_, a_1_ and a_2_ are listed in the corresponding protocol).

**Dosimetry protocol**	**Determination of k_s_**	
	**Theoretical (Formula)**	**Experimental (U_1_, U_2_, M_1_, M_2_)**
**AAPM TG-51 (1999)**	(no formula is given)	$k_{\text{s}}=\frac{U_{1}/U_{2}-1}{U_{1}/U_{2}-M_{1}/M_{2}}$
**IAEA TRS 398 (2000)**	(no formula is given)	$k_{\text{s}}=\frac{M_{1}/M_{2}-1}{U_{1}/U_{2}-1}$ or $k_{\text{s}}=a_{0}+a_{1}\frac{M_{1}}{M_{2}}+a_{2}{\left(\frac{M_{1}}{M_{2}}\right)}^{2}$
**DIN 6800-2 (2008 March)**	$k_{\text{s}}=1+\frac{\gamma +\delta \cdot D}{U_{1}}$	$k_{\text{s}}=\frac{U_{1}/U_{2}-1}{U_{1}/U_{2}-{\left[\left(M-M_{0}\right)k_{\text{p}}k_{\rho }\right]}_{1}/{\left[\left(M-M_{0}\right)k_{\text{p}}k_{\rho }\right]}_{2}}$

#### Determination of correction factor *k_P_* for the polarity of the chamber voltage

*k*_P_ can be experimentally determined by switching the polarity of the chamber voltage. The different methods for the determination of *k*_P_ according to the different protocols are shown in Table 4.

**Table 4:** Determination of the correction factors *k_p_* (where M_1_ respectively M_+_ = Monitor reading by the usual polarity, M_2_ or M_-_ = Monitor reading with opposite polarity of the chamber voltage).

**Dosimetry protocol**	**Determination of k_p_**
**AAPM TG 51 (1999)**	$k_{\text{p}}=\left(\frac{\left|M_{+}\right|+\left|M_{-}\right|}{2M}\right)/{\left(\frac{\left|M_{+}\right|+\left|M_{-}\right|}{2M}\right)}_{Co}$
**IAEA TRS 398 (2000)**	$k_{\text{p}}=\left(\frac{\left|M_{+}\right|+\left|M_{-}\right|}{2M}\right)/{\left(\frac{\left|M_{+}\right|+\left|M_{-}\right|}{2M}\right)}_{Co}$
**DIN 6800-2 (2008 March)**	$k_{p}={\left(M_{1}+M_{2}\right)/M_{1}/\left[\left(M_{1}+M_{2}\right)/M_{1}\right]}_{Co}$

However, care must be taken in case the calibration laboratory has not corrected for the polarity effect (as documented in the certification). In this case, the subsequent treatment of the polarity effect depends on the facilities available to the user, and on what beam qualities must be measured:

- If the user beam quality is the same as the calibration quality (normally Co-60) and the chamber is used at the same polarizing potential and polarity, then *k_pol_* will be the same in both cases and the user must *not* apply a polarity correction for that particular beam.
- If the user beam quality is *not* the same as the calibration quality, but it is possible to reproduce the calibration quality, then the polarity correction [*k_pol_*]_*Q*o_ that was not applied at the time of calibration must be estimated using one of the equations of Table 4 and using the *same polarizing potential and polarity* as was used at the calibration laboratory.

The polarity effect at the user beam quality, *k_pol_*, must be determined the same way using the polarizing potential and polarity adopted for routine use. A modified polarity correction is then evaluated as follows:

(3) $$k_{p}=\frac{k_{p}}{{\left[k_{p}\right]}_{Q_{o}}}$$

This is then used to correct the dosimeter readings for polarity for each beam quality Q. For the determination of [*k_p_*]_*Q*o_ measurements are generally performed with Co-60 beams. Since there is no Co-60 unit available in the authors’ department, the authors have used the smallest photon energy they have (6MV-photons) for that purpose.

#### Determination of the additional correction factor *k_r_* for cylindrical chambers specific to the German DIN protocol

This correction must always be included according to the DIN Protocol for cylindrical chambers (for photon- and electron beams). It takes into account the different position during the calibration (reference point at measuring depth) and the user’s measurement (reference point 0.5 *r_cyl_* lower than measuring depth) explicitly as a correction factor, in contrast to the other protocols, where this effect is taken into account as a perturbation factor to be applied in the calculation of the beam quality correction factor.

For the calculation of *k*_r_, the following relation is given in DIN 6800-2 (2008 March):

(4) $$k_{r}=1+\left|\delta \right|\frac{r}{2}$$

With *r_cyl_* = inner radius of the chamber cavity and *δ* = relative gradient of the dose depth curve at the reference depth during the calibration with Co-60 radiation (for Co-60-beam: *δ* = 0.006 mm^-1^). For the cylindrical chamber PTW-31013 (*r_cyl_* = 2.8 mm), *k_r_* = 1.008 is obtained.

#### Determination of correction factor *k_Q_* for the radiation quality of photons

The radiation quality must be determined before since calculated values of *k*_Q_ are provided in tables as a function of the radiation quality. The radiation quality for high-energy photon beams is characterized according to DIN 6800-2 (2008 March) and IAEA TRS 398 by the quality index Q which is specified by the tissue phantom ratio *TPR*_20,10_. This is the ratio of the absorbed doses at depths of 20 cm and 10 cm in a water phantom, measured with a constant Source-Chamber-Distance of 100 cm and a field size of 10 cm × 10 cm at the plane of the chamber. *TPR*_20,10_ can be measured directly according to its definition or by a depth dose measurement:

(5) $$Q=1.2661\cdot \frac{M(20)}{M(10)}-0.0595$$

where *M*(20) and *M*(10) are the readings at 20 cm and 10 cm depths for a field size of 10 cm × 10 cm defined at the phantom surface with an SSD of 100 cm. In the German protocol, this second method must be used.

In the American protocol TG-51, the beam quality in accelerator photon beams is specified by %dd(10)x , the percentage depth dose at 10 cm depth in a water phantom due to photons only ˜i.e., excluding electron contamination. The value of %dd(10)x is defined for a field size of 10 × 10 cm^2^ at the phantom surface at an SSD of 100 cm. Consequently, for the determination of the dose depth curve above 10 MV, a lead plate, about 1 mm thick, must be positioned between the focus and the measuring chamber. When using the same positioning as for the reference dosimetry, the dose depth curve is shifted 0.6 *r_cyl_* to take into account the displacement effect.

In DIN 6800-2 (2008 March), Table 6, IAEA TRS 398, Table 14 and AAPM TG 51, Table I, the correction factors *k*_Q_ are listed in dependence of radiation quality index *Q* for different chambers under the reference conditions. The corresponding *k*_Q_-value can be interpolated for the cylindrical chamber PTW-31013. The values of Table 6 in DIN 6800-2 (2008 March) are approximated by a polynomial of 4^th^ degree, in order to calculate the correction factor *k*_Q_ by putting any value of the quality index into the equation:

(6) $$\begin{array}{l} k_{\text{Q,PTW-31013}}\text{ = 0.584322 + 3.295307 Q }\\ -\text{ 9.246571 }{\text{Q}}^{\text{2}}\text{ + 11.275614 }{\text{Q}}^{\text{3}}\;-\text{ 5.175615 }{\text{Q}}^{\text{4}} \end{array}$$

For all protocols, a summary is given in table 6 for the determination of the radiation quality correction factor.

**Table 5:** Effective point of measurement and gradient correction for the cylindrical chamber PTW-31013.

**Radiation type**	**Process**	**DIN 6800-2 (2008 March)**	**TRS 398 (2000)**	**AAPM TG 51 (1999)**
**Photons**	Gradient correction	k_r_ = 1.008	none	none
	Effective point of measurement from the chamber axis	About 1.4 mm	none	none
**Electrons**	Gradient correction	k_r_ = 1.008	k_r_ in k_Q_	${\text{P}}_{\text{gr}}^{\text{Q}}{\text{(z}}_{\text{ref}}\text{)}$
	Effective point of measurement from the chamber axis	About 1.4 mm	About 1.4 mm	none

**Table 6:** Formalism for the determination of the quality correction factors for photon beams k_Q_(z_ref_) and Electron beams k_E_(z_ref_).

**Dosimetry- protocol**	**DIN 6800-2(2008 March)**		**IAEA 398(2000)**		**AAPM TG 51(1999)**	
**Radiation type**	**Photons**	**Electrons**	**Photons**	**Electrons**	**Photons**	**Electrons**
**Reference depth z_ref_**	10 cm	0.6*R_50_ − 0.1 cm	10 cm	0.6*R_50_ − 0.1 cm	10 cm	0.6*R_50_ − 0.1 cm
**Cylindrical-Chamber**	k_Q_° = k_Q_*k_r_	k_Q_° = k_E_'*k_E_"*k_r_	k_Q_° = k_Q_	k_Q_° = k_Q_	k_Q_° = k_Q_	k_Q_° = P^Q^_gr_*k'_R50_*k_ecal_
	k_r_ = 1 + r_cyl_ /2*δ k_Q_ from Tab. 6	k_r_ = 1 + r_cyl_ /2*δ k_E_' = f(R_50_) k_E_" from Tab. 8	k_Q_ from Tab. 14	k_Q_ aus Tab. 18	k_Q_ aus Tab. I	${\text{P}}_{\text{gr}}^{\text{Q}}\text{=}\frac{{\text{M(z}}_{\text{ref}}\text{+0}{\text{.5 r}}_{\text{cyl}}\text{)}}{{\text{M(z}}_{\text{ref}}\text{)}}$ k'_R50_ = f(R_50_) k_ecal_ aus Tab. III
	Effective point of measurement	Effective point of measurement	Chamber axis	Effective point of measurement	Chamber axis	Chamber axis
**Plane parallel Chamber**	-	k_Q_° = k_E_'*k_E_"	-	k_Q_° = k_Q_	-	k_Q_° = k'_R50_ * k_ecal_
	-	k_E_' = f(R_50_) k_E_" from Tab. 9	-	k_Q_ from Tab. 18 or k_Q_ from Tab. 19 N_w_ -> from the cross calibration	-	k'_R50_ from Gl. 20 k_ecal_ from Tab. II or k_ecal_ * N_w_^Co60^ from the cross calibration
	-	Chamber axis	-	Chamber axis	-	Chamber axis

k_Q_°: Equivalent quality correction factor

Description for the chamber positioning (Reference point):

- Reference point of the cylindrical chamber : Chamber axis with the displacement (Effective point of measurement): z_ref_ + 0.5* r_cyl_ and without displacement : chamber axis in the depth z_ref_

- Reference point of a plane-parallel chamber: on the inner window on the symmetrical axis without displacement: chamber axis in the depth z_ref_

#### Determination of correction factor *k_E_* for the radiation quality of the electrons

In all protocols the *R_50_* of the energy depth curve represents the quality index for the radiation quality. The measurement of the dose depth curves is done with a cylindrical chamber by a displacement correction of 0.5 *r_cyl_* in the direction of the focus for all protocols. This correction is not to be applied with plane parallel chambers.

The correction factor for radiation quality *k_E_* consists of a quality-specific factor (*k_E'_*) and a chamber-specific factor (*k_E"_*), which are determined separately in the DIN-Norms, whereas in the TRS 398 there is no split up.

The determination of the absorbed dose to water can be done in a quality index *R_50_* dependent reference depth *z_ref_*. The *R_50_* can be calculated from the ion depth dose curves as follows:

(7) $$R_{\text{50}}\text{ = 1.029 }\cdot R_{\text{50,ion}}\;-\text{ 0.06 in cm }$$

This equation is valid for a *R_50,ion_* < 10 cm which corresponds to an energy of < 25 MeV. From the half value depth *R_50_* , the energy dependent reference depth can be calculated as:

(8) $$z_{\text{ref}}\text{ = 0.6 }\cdot \;R_{\text{50}}\;-\text{ 0.1 in cm }$$

DIN 6800-2 defines the following equation for the determination of energy quality specific factor *k_E_'* in reference depth *z_ref_*:

(9) $$k{\text{'}}_{\text{E}}\text{(}z_{\text{ref}}\text{) = 1.106 }-\text{ 0.1312}\cdot \;R_{\text{50}}^{\text{0.214}}\;$$

Chamber quality specific factor *k_E_"* is given in the DIN 6800-2, Table 8, for different cylindrical chambers with different reference conditions in dependence of half value depth *R_50_*. These values are approximated for the cylindrical chamber PTW-31013 by a polynomial of 3^rd^ degree which deviate only of 0.3% at maximum compared to the values of the Table 8.

(10) $$\begin{array}{c} k_{E}^{"}{{\text{(z}}_{ref}\text{)}}_{\text{PTW-31013}}\text{ = 0.947120 + 0.007313 }R_{\text{50}}\;\\ -\text{ 0.000352 }R_{50}^{2}\text{ + 0.000006 }R_{50}^{3}\; \end{array}$$

Chamber specific factor *k_E_"* for plane parallel chambers equals the inverse value of the chamber specific perturbation factor *p_Co_* for Co-60-beams, which is, according to DIN 6800-2 (2008 March), Table 9:

(11) $$k_{E}^{"}{{\text{(z}}_{ref}\text{)}}_{\text{PTW-Roos}}\text{ = 0.981}$$

Therefore, the time-consuming cross calibration measurement for the determination of *k_E_"* for all known chambers is not necessary in DIN 6800-2 (2008 March). It is only necessary for the introduction of new types of chambers.

According to TRS 398, the product *k_E_'* * *k_E_"* (= *k_E_*, in TRS as *k_Q_* described) for diverse cylindrical and plane parallel chambers in TRS 398, Table 18, is listed as a function of the radiation quality *R_50_* for the reference depth and can be found by interpolation for the cylindrical chamber PTW-31013. The IAEA proposes, on the other hand, to perform a cross calibration for plane parallel chambers by using a cylindrical chamber instead of using the inaccurate values of Table 18. The authors have performed the cross calibration with the highest recommended energy of each accelerator (14 and 21 MeV-Electrons). Thereby, the calibration factor of the Ross-chamber will be newly determined. After that, the radiation quality factor of the Roos-chamber can be evaluated with the help of IAEA TRS 398, Table 19. The exact procedures are presented in literature [3, 6].

According to AAPM TG-51, the quality correction factor *k*_E_ for electrons as one of the constituent correction factors in equation 1 is substituted by:

(12) $$k_{\text{E}}=P_{gr}^{Q}\cdot {k^{\prime }}_{R_{50}}\cdot k_{ecal}$$

*k_ecal_* is the chamber type dependent calibration factor *N*_w_ for Co-60- beam transformed to the reference radiation quality *R*_50_ = 7.5 cm. ${k^{\prime }}_{R_{50}}$ is the calibration factor of this energy converted to the actual radiation quality *R*_50_. $P_{gr}^{Q}$ is an additional component of *k*_E_ and is the gradient correction factor in an electron beam that is dependent on the ionization gradient at the point of measurement. For cylindrical chambers, $P_{gr}^{Q}$ is a function of the radius of the cavity; it is equal to 1 for plane-parallel chambers. *k_ecal_* is given in the AAPM-Protocol for different chambers. ${k^{\prime }}_{R_{50}}$ can be calculated for Farmer-type cylindrical chambers through an approximate formula given in the dosimetry protocol for reference depth *z_ref_* [5, 7]. A cross calibration for plane parallel chambers is also recommended by the AAPM.

A summary of the formalism for the determination of the radiation quality correction concerning electrons is given for all three protocols in Table 6.

## RESULTS AND DISCUSSION

For the photons and electrons, the deviation of the measuring values for both cylindrical chambers amounts about ± 0.3%, whereas for Roos chambers it is about ± 0.2%. These values are deduced from multiple measurements followed by a calculated average value with every chamber type for each protocol.

**Figure 1:**
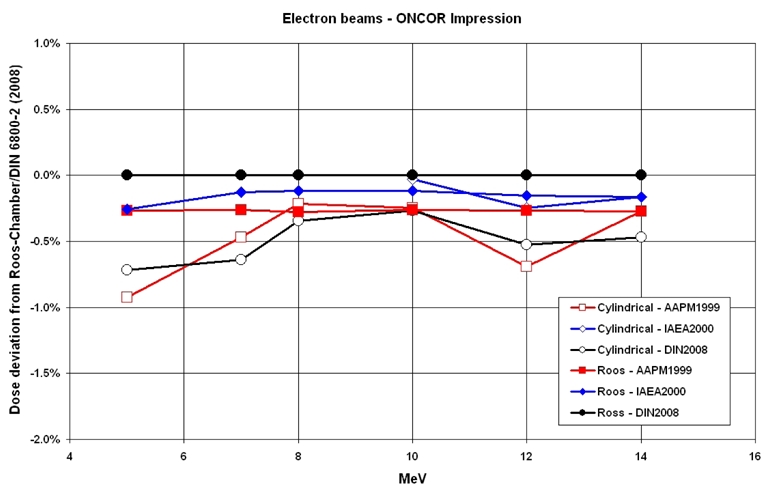
Comparison of the absorbed dose ratios in photon beams from the linear accelerators ONCOR Impression and ONCOR Avantgarde.

In Figure 1, the deviations of the dose values for photon beams measured with a cylindrical chamber are presented with respect to DIN 6800-2 (2008 March).

**Figure 2:**
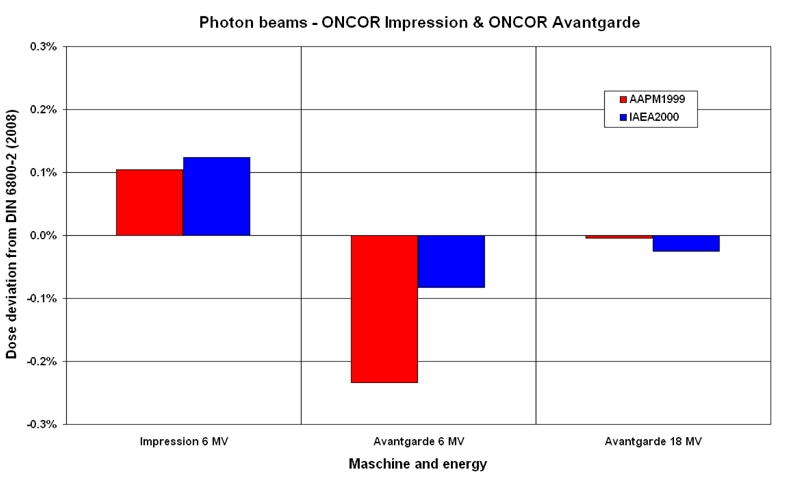
Comparison of the absorbed dose ratios as a function of electron energy from the linear accelerator ONCOR Impression.

**Figure 3:**
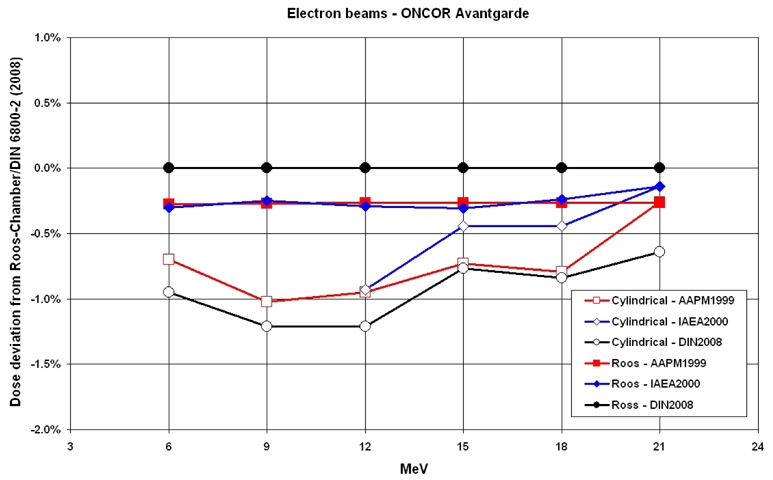
Comparison of the absorbed dose ratios as a function of electron energy from the linear accelerator ONCOR Avantgarde.

Figures 2 and 3 show the deviations of the dose values for electron beams, which are normalized to the values of the Roos-chamber according to DIN 6800-2 (2008 March), for both chamber types as a function of the energy for all three protocols. The measurements are done for both photon and electron beams with cylindrical chambers whereas the plane parallel chambers are only used for the electron beams.

Figure 1 shows the deviations for photon beams for both linear accelerators. According to AAPM and IAEA protocols from the values from DIN 6800-2 (2008 March), the deviations rise to a maximum of 0.23%. The DIN was adapted to the IAEA. As expected, the deviation of the IAEA from the DIN (2008) reaches an average of a max. of about 0.1%.

Figures 2 and 3 show the deviations by the electron beams, each for one accelerator. The deviations for all chambers are given in relation to the values of the Roos-chamber according to protocol DIN 6800-2 (2008 March). The measuring values of all chambers according to all protocols are generally smaller than the values of the Roos-chamber according to DIN 6800-2 (2008 March). The largest deviation is about -1.3%. As observed, the deviation of the dose values between the cylindrical and the plane parallel chambers varies between 0.5 to 1.3% according to DIN 6800-2 (2008 March).

For the electron beams, it shows the tendency that the measuring values of the Roos chamber deviate from each other for only a maximum of about 0.3% according to TRS 398 and AAPM in reference to DIN 6800-2 (2008 March), whereas the corresponding values of the cylindrical chamber are of a max. of about 1.0 %.

The results following DIN 6800-2 are checked with TLD inter-comparison by the MTK. For photon beams, a deviation of max. 1.8% and for electron beams of max. 2.0% are found between the TLD measurements and the measurements with chambers according to DIN 6800-2 (2008 March).

In an earlier work, the authors compared protocols DIN 6800-2 (1997), IAEA TRS 398 and AAPM TG 51 for the linear accelerators Siemens Mevatron M6300 and M7445. In that work, the deviations of the two other protocols in relation to DIN 6800-2 (1997) showed 1% for photons and 1.6% for electrons [7]. In contrast to that work the current deviations are decreased by more than a half for photon beams whereas the values for the electrons progressed a little.

The uncertainties in the measured values consist of a series of independent, single uncertainties. The total uncertainty is calculated from the geometrical sum of the individual uncertainties. In the protocol AAPM TG 51 (1999) no uncertainties are given for the determination of the correction factors for the radiation quality. In contrast to that, a discussion on the uncertainty measurements of the radiation quality corrections is found in DIN 6800-2 (2008 March) and IAEA TRS-398. By the other influencing variables, the uncertainties were estimated by us or the manufacturer’s information was considered.

For the determination of dose for photon and electron beams with cylindrical chambers, the total standard uncertainties is estimated according to TRS 398 a maximum of 1.25% and a maximum of 1.42% according to DIN 6800-2 (2008 March), whereas for electron beams with Roos chamber 0.96% and 1.50% according to TRS 398 and DIN 6800-2 (2008) is found, respectively. The influence quantities which contribute to an important part to the total uncertainty are presented in Table 7 [8].

**Table 7:** Influence quantities and their contributions to total uncertainties.

**Influence quantities**	**Source**	**Cylindrical chamber Photon beams**	**Cylindrical chamber Electron beams**	**Roos- chamber Electron beams**
**N_w_**	DIN 6800-2 (2008)	0.45	0.45	0.45
**Depth of measurement**	Estimation	0.1	0.1	0.1
**SSD**	Estimation	0.1	0.1	0.1
**Leakage current**	Manufacturer’s figure	0.2	0.2	0.2
**k_P_**	DIN 6800-2 (2008)	0.1	0.1	0.1
**k_S_**	DIN 6800-2 (2008)	0.1	0.1	0.1
**k_ρ_**	DIN 6800-2 (2008)	0.17	0.17	0.17
**k_Q_ or k_E_**	IAEA TRS 398	1.0	0.9	0.6
**k_Q_ or k_E_**	DIN 6800-2 (2008)	1.0	1.2	1.3
**Dosimeter reading**	Manual PTW-UNIDOS	0.5	0.5	0.5
**Long-term stability Dosimeter/Year**	Manual PTW-UNIDOS	0.1	0.1	0.1
**Total uncertainty (TRS 398)**		**1.25**	**1.17**	**0.96**
**Total uncertainty (DIN 6800-2)**		**1.25**	**1.42**	**1.50**

## CONCLUSION

DIN 6800-2 was widely revised and almost adapted to the IAEA TRS-398. While in the IAEA and the AAPM , a cross calibration measurement for plane parallel chambers with a cylindrical chamber is recommended, an experimental calibration factor for the different plane parallel chambers is given in DIN 6800-2 (2008 March) so that the relative inaccurate individual cross calibration measurement is spared for the user. In DIN 6800-2 (2008 March), an analysis of the measurement uncertainties is discussed for the first time.

In connection with the TRS 398, measurements of electron beams are now also done in *z_ref_* according to DIN 6800-2 (2008). Furthermore, only the *R_50_* is used in contrast to the old DIN 6800-2 (1997) where both *R_50_* and *R_p_* were used for the characterisation of the electron radiation quality.

While in the DIN protocol no limits are mentioned by the use of a cylindrical chamber for electron energy lower than 10 MeV, the IAEA TRS 398 and AAPM do not recommend the use of the cylindrical chamber for electron beams with energy below 10 MeV. The IAEA gives no correction factors for that radiation quality whereas there exits a possibility of calculation through a formula according to AAPM.

The deviation between the three protocols is less than 0.23% for the photons, and stays within a maximum of 1.2% for the electrons. A measuring point displacement (effective point of measurement) is done in the DIN by the cylindrical chambers for photon beams which is considered by the correction factor kr, whereas according to IAEA and AAPM no displacement of the measuring point is necessary. However, according to AAPM, radiation quality factors can be explicitly considered through the gradient factor. In the IAEA protocol, the gradient correction is contained in the radiation quality factor for the electrons. Results showed that the deviation for the electron beams between the protocols DIN 6800-2 (2008), IAEA and AAPM in reference to the Roos-chamber is relatively low.

The authors’ experience shows that the time required for the clinical dosimetry is almost the same as for all three protocols. The advantage of DIN 6800-2 (2008 March) lies in the following points: there is no need for cross calibration measurement for plane parallel chambers as given in the protocol as well as a clear overview of all important formulas and parameters needed for the dosimetry. The IAEA and AAPM and the DIN give parameters for a number of the existing chambers on the market.

For the introduction of new chambers, the corresponding parameters must be determined by other ways, for example by a time expensive cross calibration formalism or through the manufacturer company.

In the past the different protocols were developed differently from time to time; fortunately today, we have achieved a good convergence in the concepts and methods.
